# The Influence of Fed State Lipolysis Inhibition on the Intraluminal Behaviour and Absorption of Fenofibrate from a Lipid-Based Formulation

**DOI:** 10.3390/pharmaceutics14010119

**Published:** 2022-01-04

**Authors:** Marlies Braeckmans, Joachim Brouwers, Danny Riethorst, Cécile Servais, Jan Tack, Patrick Augustijns

**Affiliations:** 1 Drug Delivery and Disposition, KU Leuven, Gasthuisberg O&N II, Herestraat 49-Box 921, 3000 Leuven, Belgium; marlies.braeckmans@kuleuven.be (M.B.); joachim.brouwers@kuleuven.be (J.B.); driethorst@gmail.com (D.R.); 2 Galephar M/F Research Center, 6900 Marche-en-Famenne, Belgium; cserv@galephar.be; 3 Translational Research Center for Gastrointestinal Disorders, TARGID, KU Leuven, 3000 Leuven, Belgium; jan.tack@kuleuven.be

**Keywords:** small intestine, drug absorption, lipolysis, lipid-based formulation, fenofibrate, aspiration

## Abstract

The bioavailability of lipophilic drugs may or may not be increased when administered with food due to increased solubilisation in fed state gastrointestinal (GI) fluids. The in vivo interplay between drug solubilisation, lipid phase digestion and drug absorption is complex and remains poorly understood. This study aimed to investigate the role of fed state GI lipolysis on the intraluminal behaviour and absorption of fenofibrate, formulated as the lipid-based formulation Fenogal. Therefore, a crossover study was performed in healthy volunteers using orlistat as lipase inhibitor. Fenofibrate concentrations were determined in the proximal jejunum and linked to simultaneously assessed systemic fenofibric acid concentrations. Inhibition of lipolysis by orlistat resulted in a faster onset of absorption in 4 out of 6 volunteers, reflected by a decrease in systemic T_max_ between 20 and 140 min. In addition, the increase of undigested lipids present in the small intestine upon orlistat co-administration sustained drug solubilisation for a longer period, resulting in higher fenofibrate concentrations in the jejunum and improved absorption in 5 out of 6 volunteers (median AUC_0–8h_ 8377 vs. 5832 μM.min). Sustaining drug solubilisation in the lipid phase may thus contribute to the absorption of lipophilic drugs. More research into the different mechanisms underlying lipophilic drug absorption from fed state media at different levels of digestion is warranted.

## 1. Introduction

To this day, it remains a challenge to develop novel formulations enhancing the oral bioavailability of lipophilic drugs. Often, these drugs display a large intra- and interindividual variability due to their poor solubility in the aqueous luminal environment of the gastrointestinal (GI) tract, which is influenced by the presence of solubilising structures, such as (mixed-)micelles, vesicles and lipid droplets. In this respect, a variable food effect can often be observed depending on the prandial state at the time of drug intake and the characteristics of the meal [1,2]. The composition of the fed state intraluminal GI environment is highly dynamic due to food digestion. Triacylglycerols (TAGs) break down into diacylglycerols (DAGs), monoacylglycerols (MAGs) and free fatty acids (FFAs), which in turn are incorporated in different types of colloidal structures with the aid of bile salts. Hence, the solubilizing capacity of the small intestinal contents for lipophilic drugs is usually higher but also more variable in the fed state compared to the fasted state.

Even though drug molecules solubilised by lipid colloids may not be readily available for uptake, plenty of data suggest a beneficial effect of lipids (either dietary- or formulation-derived) on the absorption of a range of lipophilic drugs, which also led to the development of lipid-based formulations (LBFs) [3]. During lipid digestion, the intraluminal ultrastructure transitions from lipid droplets and larger lipid-rich colloids to smaller (mixed-)micelles, which causes changes in solubilizing capacity for lipophilic drugs. This might be beneficial for drug absorption. In case of LBFs, for instance, it has been shown that drug supersaturation, a potential driving force for absorption, can be induced at different moments during LBF processing in the GI tract: (i) upon dispersion of the LBF in the gastric environment and dilution of water miscible co-solvents or surfactants present in the LBF, (ii) during digestion of lipids and/or surfactants within the formulation, (iii) upon dilution of lipid-rich colloidal species by bile and (iv) upon absorption of FFAs present in (mixed-)micelles solubilising the compound [4]. In theory, similar mechanisms may also contribute to the mechanism behind the positive food effect that is often observed for lipophilic drugs by altering the solubilizing capacity of the small intestinal intraluminal environment. 

The effects of in vivo processing of meal- or formulation-derived lipids on drug and formulation behaviour are complex and research is currently limited to ex vivo and in vitro analysis. Therefore, the present study aims to investigate the influence of lipid digestion in the human GI tract on the uptake of the cholesterol- and TAG-lowering agent fenofibrate (log P 5.21, non-ionisable, BCS class II). Its high lipophilicity and poor aqueous solubility cause a low bioavailability after oral intake. Over the years, different formulation strategies have been used to overcome the poor oral bioavailability of fenofibrate, such as particle size reduction and LBFs. It has been reported that the intake of a high-fat meal resulted in increased duodenal concentrations of fenofibrate when administered as micro- or nanoparticles (i.e., Lipanthyl or Lipanthylnano). However, this increase did not translate into a higher systemic exposure in postprandial conditions, probably due to micellar encapsulation, showing that intestinal solubilisation does not necessarily lead to improved systemic absorption [5]. On the other hand, fenofibrate is on the market as the LBF Fenogal. Fenogal contains homogeneously dispersed fenofibrate (200 mg) within a lipid excipient mixture (Gelucire 44/14) and can be classified as a class IIIb formulation according to the Lipid Formulation Classification System (LFCS) [6]. Gelucire 44/14 is a semi-solid self-emulsifying lipid excipient, composed of C8-C18 MAGs, DAGs and TAGs (solubilisation), C8-C18 mono- and diesters of PEG-32 (surfactants) and free PEG-32 (co-solvent), which forms a finely dispersed microemulsion when in contact with the aqueous environment of the GI tract [7]. Although elimination of a food effect can be observed in vivo for some compounds formulated as LBFs [8,9,10], Fenogal still needs to be taken with a meal to enhance the oral bioavailability of fenofibrate [6]. In theory, both acylglycerols and PEG esters are substrates for digestive enzymes. Fernandez et al. investigated the activity of different digestive lipases on Gelucire 44/14 in vitro [11]. During simulation of gastric lipolysis, the C8-C18 TAGs were rapidly hydrolysed into MAGs. Although human pancreatic lipase (HPL) exerted only low activity, Gelucire 44/14 was shown to be a good substrate for other digestive lipases, such as carboxyl ester hydrolase (CEH) [7]. Thus, the composition of Gelucire 44/14 changes upon dispersion and digestion, which can result in a reorganisation of the microemulsion system over time. The obtained MAGs and PEG monoesters might be included in colloidal structures, aiding in the solubilisation and transport of the drug toward the unstirred water layer in vivo. Inhibiting the naturally occurring process of lipid digestion in the fed state GI tract might therefore have a detrimental effect on the release and absorption of a compound such as fenofibrate from an LBF comprised of Gelucire 44/14. In addition, impaired GI lipolysis will result in the presence of a higher amount of intact meal TAGs and a lower amount of lipolysis products in the small intestine, leading to a different intraluminal ultrastructure, which can also affect drug solubilisation and absorption.

The fate of fenofibrate in the fed state small intestine after oral administration of Fenogal has not been examined so far in vivo. To evaluate the role of GI lipolysis on the intraluminal behaviour and systemic absorption of fenofibrate from Fenogal in the fed state, orlistat (Xenical) was co-administered. Orlistat (tetrahydrolipstatin) covalently blocks the active site of human gastric (HGL) and pancreatic lipases [12,13]. Orlistat is reported to inhibit both HGL (46.6–91.4% enzyme inhibition) and HPL (51.2–82.6% enzyme inhibition), after intake of solid and liquid meals [14]. In addition, human carboxyl-ester lipase (HCEL) is reversibly inhibited [15,16]. As orlistat reduces the formation and absorption of lipid digestion products, such as MAGs and FFAs, it is on the market to treat obesity. Ironically, orlistat is very lipophilic and requires administration during a (high-fat) meal for its action. Due to its high lipophilicity, orlistat acts locally in the GI tract and the systemic uptake is negligible [17]. 

In brief, the aim of this study was to examine the importance of in vivo lipid digestion on the absorption of the lipophilic model compound fenofibrate from the LBF Fenogal in fed state conditions. For this purpose, Fenogal was administered to healthy volunteers after a liquid meal, with and without orlistat (Xenical) as lipase inhibitor. Dissolved fenofibrate concentrations in the small intestine (proximal jejunum) and systemic fenofibric acid concentrations (active metabolite) were assessed simultaneously in an attempt to evaluate the effect of inhibiting GI lipolysis on both the intraluminal behaviour and the systemic appearance of the drug.

## 2. Materials and Methods

### 2.1. Chemicals

Fenofibrate and fenofibric acid were obtained from APIchem (Shanghai, China) and ABCR GmbH & Co (Karlsruhe, Germany), respectively. Carbamazepine was acquired from PharmInnova (Waregem, Belgium). NaOH and HCl were purchased from Sigma Aldrich (St. Louis, MO, USA). Acetonitrile, methanol, dimethylsulfoxide (DMSO) and dichloromethane were ordered from Acros Organics (HPLC grade; Geel, Belgium). Sodium acetate trihydrate (NaOAc.3H_2_O) was obtained from VWR Belgium (Haasrode, Belgium) and acetic acid (HOAc) from Chem-Lab NV (Zedelgem, Belgium). Water was purified using a Maxima system (Elga, Ltd., High Wycomb Bucks, UK). Dry ice was acquired from Ijsfabriek Strombeek N.V. (Meise, Belgium).

### 2.2. Clinical Study

#### 2.2.1. Study Medication

Fenogal Lidose capsules containing 200 mg of fenofibrate (Laboratoires SMB N.V., Brussel, Belgium) and Xenical capsules containing 120 mg of orlistat (Cheplapharm Arzneimittel GmbH, Greifswald, Germany) were both ordered from the hospital pharmacy of the University Hospitals Leuven (UZ Leuven, Leuven, Belgium).

#### 2.2.2. Study Setup

An exploratory crossover study was performed in 6 healthy volunteers (HV; 1 man, 5 women; aged between 19 and 55 years) in the fed state. Initially, eight healthy volunteers were included, but two dropped out during or after completion of one test condition (data not included in the analysis). Exclusion criteria were (potential) pregnancy, history of GI pathology and/or illness at the time of the study. The study procedure was in accordance with the Declaration of Helsinki and approved by the Ethics Committee Research UZ/KU Leuven (EC reference number S60080) and by the Federal Agency for Medicines and Health Products (FAMHP; EudraCT reference number 2016-005248-41). All volunteers provided written informed consent before participating in the clinical study.

**Figure 1:**
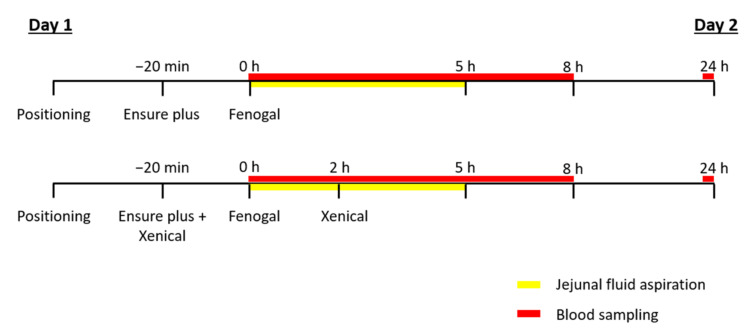
Schematic representation of the clinical study wherein Fenogal was administered in the fed state, with and without the administration of Xenical.

After an overnight fast of at least 10 h, during which only water was allowed, a customized 2-channel aspiration catheter (outer diameter 4.6 mm, inner diameter of lumina 1.3 and 2.9 mm, body length 200 cm; MUI Scientific, Mississauga, ON, Canada) was positioned through the nose or mouth in the proximal jejunum (approximately 70 cm from the pylorus). If needed, drinking of tap water was allowed to facilitate the intubation. Positioning of the catheter was checked with fluoroscopy. In addition, an intravenous catheter was placed for repeated blood sample collection. Volunteers were asked to sit in a semi-supine position during the experiment. The following conditions were tested in random order on different days with a minimum washout period of seven days (Figure 1):

— Oral administration of one capsule of Fenogal (200 mg of fenofibrate) in the fed state.
— Oral administration of one capsule of Fenogal (200 mg of fenofibrate) and two capsules of Xenical (2 × 120 mg of orlistat) in the fed state.

To simulate the fed state, volunteers were asked to drink 400 mL of Ensure Plus nutrient shake (Abbott Laboratories B.V., Zwolle, The Netherlands) 20 min prior to the oral intake of Fenogal with 240 mL of water. The energy value of this liquid meal is 2528 kJ/600 kcal (proteins 17%, carbohydrates 54% and lipids 29%). To ensure lipase inhibition throughout the experiment, Xenical was administered twice, once together with Ensure Plus and a second time 2 h after administration of Fenogal with sufficient water to swallow the capsule (<100 mL).

After oral administration, intestinal fluids were aspirated in the fed state for 5 h. Samples were taken every 20 min during the first hour, followed by every 15 min for the next 4 h. The volume of the aspirated samples was kept as small as possible (<3 mL per sample). In parallel, venous blood samples were collected in heparinized tubes (BD Vacutainer systems, Novolab NV, Geraardsbergen, Belgium) every 30 min during the first 2 h, followed by every 20 min for the next 4 h and every 30 min until 8 h. A final venous blood sample was collected 24 h after administration of Fenogal.

### 2.3. Sample Analysis

#### 2.3.1. Jejunal Samples

Immediately after sampling, the pH of the jejunal aspirates was measured (Hamilton Knick Portamess, Bonaduz, Switzerland). Subsequently, part of the sample was centrifuged (20,817× *g*, 5 min; Microcentrifuge 5424, VWR International), and the supernatant was diluted with MeOH:H_2_O (50:50 *v*/*v*) if no lipid layer was present on top of the sample. In case a separated lipid layer was present after centrifugation, the full supernatant was transferred into a new microcentrifuge tube, vortexed and diluted with MeOH:H_2_O (50:50 *v*/*v*) to determine the amount of fenofibrate in the combined lipid and micellar layer (total sample). To determine fenofibrate concentrations in the micellar layer, the supernatant was centrifuged a second time (20,817× *g*, 30 min, 37 °C) on the day of analysis, and the lipid layer was discarded. Subsequently, part of the micellar layer was diluted with MeOH:H_2_O (50:50 *v*/*v*). All intestinal samples were kept on dry ice until the end of the experiment, after which they were stored at −20 °C pending analysis.

Fenofibrate was analysed using an HPLC system consisting of an Alliance 2695 separations module and a Novapak C18 column under radial compression (Waters, Milford, MA, USA). After centrifugation of the processed samples (20,817× *g*, 5 min), 50 µL of the supernatant was injected. Fenofibrate was eluted isocratically, using MeOH:25 mM sodium acetate buffer pH 3.5 (80:20 *v*/*v*) as the mobile phase. The retention time of fenofibrate was 12.3 min. After elution of fenofibrate, the column was rinsed using ACN:H_2_O (95:5 *v*/*v*) for 2 min, followed by MeOH:H_2_O (25:75 *v*/*v*) for 2 min. Subsequently, the column was re-equilibrated with the initial mobile phase for 5 min. Fenofibrate was detected using UV detection at a wavelength of 287 nm (Waters 2487 UV Detector). Calibration curves were made in MeOH:H_2_O (50:50 *v*/*v*). Linearity was demonstrated over the range of 200 to 0.0977 µM. All intestinal samples were diluted to fit this range. Quality control samples were prepared in MeOH:H_2_O (50:50 *v*/*v*) containing 100, 10 and 1 µM of fenofibrate and included on the days of analysis. Concentrations of fenofibrate could be determined precisely (RSD ≤ 1.3%) and accurately (relative bias < 4%).

#### 2.3.2. Plasma Samples

Venous blood samples were kept on ice before being centrifuged at the end of the experiment (2880× *g*, 10 min, 4 °C; Centrifuge 5804R, Eppendorf, Hamburg, Germany). The resulting plasma samples were stored at −20 °C pending analysis. Fenofibric acid (active metabolite) was extracted from plasma as described by Hens et al. [5]. Briefly, 100 μL of the internal standard solution (20 μM carbamazepine in 1 M HCl) was added to 500 μL of plasma. Subsequently, 400 μL of 1 M HCl was added, and the mixture was vortexed (±10 s). After extraction with 6 mL of dichloromethane (vortexed for 1 min and centrifugation (2880× *g*, 15 min, 4 °C)), the upper layer was discarded and the organic layer evaporated to dryness under a gentle stream of air. Subsequently, the residue was dissolved in 1 mL of methanol. After evaporation, the sample was reconstituted using 200 µL of mobile phase and centrifuged (20,817× *g*, 5 min). Subsequently, 50 µL of the supernatant was injected into the HPLC system described above. Carbamazepine and fenofibric acid were detected at a wavelength of 287 nm. The mobile phase consisted of ACN:25 mM sodium acetate buffer pH 3.5 (50:50 *v*/*v*). After 3 min, ACN was increased to 60%. After 10 min, the column was rinsed using ACN:H_2_O (90:10 *v*/*v*) during 2 min followed by 1 min of ACN:H_2_O (25:75 *v*/*v*). The column was reconditioned with the initial mobile phase for 5 min. A flow rate of 1 mL/min generated retention times of 4.3 and 7.3 min for carbamazepine and fenofibric acid, respectively. Calibration curves were made by spiking blank plasma with fenofibric acid and were linear between 200 and 0.0977 µM. Quality control samples (100, 10 and 1 µM) were prepared in the same way and included on the days of analysis resulting in accuracy errors of less than 8%. 

### 2.4. Data Analysis

Drug concentration-time profiles are presented individually. Systemic pharmacokinetic parameters (i.e., T_max_, C_max_, AUC_0–8h_) were calculated using Graphpad Prism (version 8.0.1; Graphpad Software Inc, La Jolla, CA, USA). Since this study followed an exploratory design, no hypothesis testing was performed.

## 3. Results and Discussion

**Figure 2:**
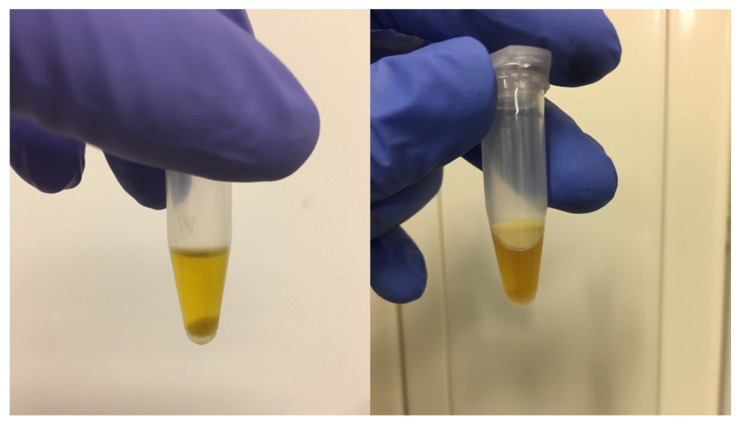
Representative examples of intestinal fluid samples aspirated from the jejunum after intake of Fenogal in the fed state without (**left**) and with (**right**) co-administration of Xenical (orlistat) as lipase inhibitor. After centrifugation, a lipid layer could be clearly distinguished on top of the aqueous micellar layer after orlistat co-administration (**right**) but was absent from jejunal fluids in the fed state without lipolysis inhibition.

A crossover study in 6 volunteers was performed to evaluate the effect of fed state lipolysis inhibition on fenofibrate absorption after oral administration of the LBF Fenogal. The partial inhibition of lipid digestion by orlistat could be visually observed after aspiration and subsequent centrifugation of the fed state jejunal fluids (Figure 2). In the presence of orlistat, a lipid layer could be observed on top of multiple centrifuged fluids from the different volunteers. Such a lipid layer was absent in the jejunal fluids aspirated in the fed state without orlistat, indicating a fast and more complete lipolysis of the liquid meal in the stomach and proximal part of the small intestine. Imaging of duodenal fed state human intestinal samples in a previous study revealed that such a lipid layer contains lipid droplets and other colloidal structures (i.e., vesicles). These structures are absent from the rest of the sample, which only contains micellar structures [18]. Hence, the ultrastructure of intestinal samples is clearly affected by co-administration of orlistat, possibly affecting drug solubilisation and absorption. 

**Figure 3:**
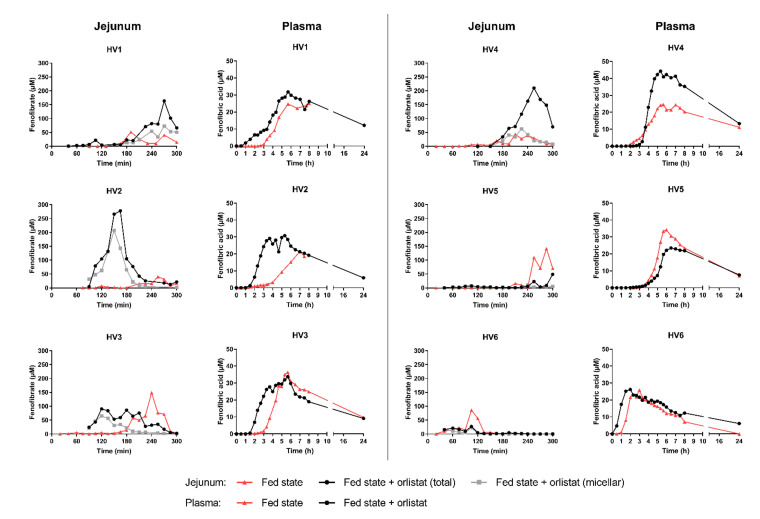
Individual concentration-time profiles of fenofibrate in the jejunum and fenofibric acid in the systemic circulation after intake of Fenogal (200 mg fenofibrate) in the fed state with (●) and without orlistat (▲). When digestion was inhibited with orlistat, a lipid layer was present upon centrifugation of multiple intestinal samples, hence concentrations in both total sample (lipid + micellar layer; ●) and micellar layer (■) were determined in the fed state with orlistat.

Since the appearance of fenofibrate in the jejunum and the systemic uptake of the active metabolite fenofibric acid were highly variable between the different volunteers, individual concentration-time profiles are presented in Figure 3. Jejunal concentration-time profiles for fenofibrate are presented next to the corresponding plasma concentration-time profiles. Based on the systemic profiles, lipolysis inhibition appeared to affect both the onset and the extent of absorption.

### 3.1. Effect on the Onset of Absorption

In the fed state without orlistat co-administration, fenofibrate could be detected in the jejunal aspirates only after a lag time of at least 3 h in 5 out of 6 volunteers, most likely reflecting a prolonged gastric emptying time in the fed state (Figure 3). Consequently, the systemic appearance of fenofibric acid was delayed in these volunteers (lag time to reach 5% of C_max_ > 140 min; Table 1), resulting in a systemic T_max_ of at least 340 min. Only in HV6, fenofibrate could already be detected in the jejunum 40 min after intake, suggesting a fast gastric emptying of the drug. Measured jejunal concentrations in this volunteer were relatively low, but higher concentrations might be reached at more distal segments compared to the sampling site due to the fast transit. The early small intestinal appearance of fenofibrate in HV6 resulted in a faster onset of absorption (systemic lag time of 60 min) and a reduced T_max_ (180 min). Overall, the median systemic lag time to reach 5% of the systemic C_max_ amounted to 170 min, with a median T_max_ of 350 min (Table 1). According to the Summary of Product Characteristics (SmPC), Fenogal is reported to reach C_max_ after 4 to 5 h when taken with a meal.

**Figure 4:**
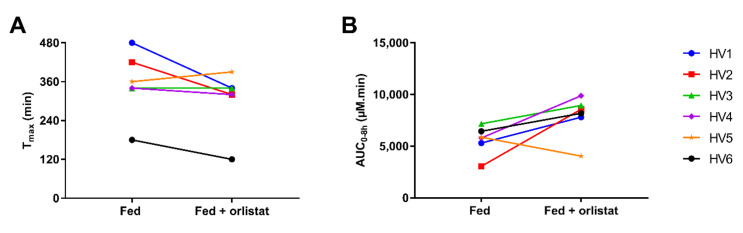
Comparison of the individual systemic pharmacokinetic parameters (**A**) T_max_ and (**B**) AUC_0–8h_ after intake of Fenogal (200 mg fenofibrate) in the fed state (left) and fed state with orlistat (right).

When orlistat was administered with the meal 20 min prior to Fenogal, a faster onset of appearance of fenofibric acid in the systemic circulation was observed in four healthy volunteers (i.e., HV1, HV2, HV3 and HV6; Figure 3 and Table 1). In three out of these four volunteers (i.e., HV1, HV2 and HV3), the faster onset of absorption was associated with a faster detection of fenofibrate in the jejunum. Overall, the median lag time to reach 5% of the systemic C_max_ was 120 min compared to 170 min without orlistat (Table 1). In HV4 and HV5, orlistat administration did not result in a faster onset of fenofibrate absorption. In HV5, the onset was comparable between both conditions, probably due to slow gastric emptying of the formulation as can be observed in the jejunal concentration-time profiles. Gastric emptying of the meal was also delayed in both conditions in this volunteer, as further discussed in Section 3.2. In HV4, fenofibric acid appeared in the systemic circulation one hour earlier in the fed state compared to the fed state with orlistat. This observation was not accompanied by a faster appearance of fenofibrate in the jejunum, which was comparable in both conditions. Still, in 4 out of 6 healthy volunteers, the systemic T_max_ decreased (Figure 4).

It has been reported in literature that orlistat can affect the systemic T_max_ by altering the gastric emptying rate [19]. The gastric emptying rate is regulated by a feedback inhibition caused by the presence of nutrients in the small intestine and is particularly dependent on the lipolysis of TAGs to FFAs. For instance, the gastric emptying of alcohol was accelerated after ingestion of fat together with orlistat [20]. Intake of 120 mg of orlistat resulted in higher initial blood glucose and plasma insulin concentrations caused by faster gastric emptying of a test meal [21]. Similar conclusions were made in another study, in which the gastric emptying of oil and glucose occurred faster after orlistat administration compared to the control [22]. In addition, an increase in the number of antral and duodenal pressure waves together with stimulation of antropyloroduodenal pressure-wave sequences has been observed as a response to an intraduodenal fat infusion containing orlistat [23]. Hence, it seems likely that a faster gastric emptying, possibly combined with more postprandial contractions, caused the faster small intestinal appearance of fenofibrate and subsequent absorption, which was observed in most volunteers upon orlistat co-administration.

### 3.2. Effect on the Extent of Absorption

In addition to affecting the onset of absorption and the systemic T_max_, the partial inhibition of GI lipolysis by orlistat altered the extent of fenofibric acid exposure. In 5 out of 6 volunteers (not HV5), AUC_0–8h_ increased after intake of orlistat (Figure 4), resulting in an increase in median AUC_0–8h_ from 5832 to 8377 µM.min (Table 1). In these five volunteers, a lipid layer was observed after centrifugation of the intestinal aspirates after intake of orlistat (illustrated in Figure 2). Therefore, fenofibrate concentrations were determined in both the micellar layer and the total sample (micellar + lipid layer) when orlistat was co-administered (Figure 3). Higher fenofibrate concentrations were generally measured in the total sample compared to the micellar layer, indicating the increased solubilisation of fenofibrate in the presence of a lipid layer. This is in accordance with Riethorst et al., who studied ex vivo the solubility of fenofibrate in aspirated duodenal fed state fluids with and without inclusion of the lipid layer [24]. 

In three volunteers (i.e., HV1, HV2 and HV4), the higher systemic AUC_0–8h_ upon partial lipolysis inhibition by orlistat was accompanied by substantially higher fenofibrate concentrations in the small intestinal aspirates, both in the total sample and micellar layer. Interestingly, the effect of orlistat on jejunal fenofibrate concentrations was most pronounced in HV2 and HV4, corresponding to the largest increase in systemic AUC_0–8h_ (2.8- and 1.7-fold, respectively). In HV3, orlistat did not have a clear effect on jejunal fenofibrate concentrations, despite a slight increase in systemic AUC_0–8h_. In HV6, gastric emptying and intestinal transit of the formulation were very fast in both test conditions, making it impossible to adequately detect the influence of lipase inhibition at the sampling site in the proximal jejunum. 

Only in HV5, the systemic C_max_ and AUC_0–8h_ slightly decreased upon co-administration of orlistat. Most likely, this deviating observation is linked to the slow gastric emptying of Fenogal and the liquid meal in both test conditions. Only at the end of the 5 h sampling period, fenofibrate appeared at the jejunal sampling site and aspirates had the appearance of fed state samples. In contrast to the other volunteers, co-administration of orlistat did not result in a visually clear lipid layer on top of the aspirates. It seems plausible that the absence of a clear orlistat effect on lipid digestion in HV5 is related to the deviating effect on the systemic exposure to fenofibric acid, stressing the importance of fenofibrate solubilisation in the lipid layer to increase absorption.

In this study, the increased amount of intact lipids after orlistat co-administration clearly assisted in the solubilisation of fenofibrate in the small intestine. It is still uncertain how and to what extent drug molecules solubilised in the lipid droplets and large colloidal structures of the lipid layer contribute to systemic absorption. Currently, more evidence exists that solubilised drugs are absorbed via the free concentration that exists in rapid equilibrium with the solubilised reservoir, rather than via direct interaction of the solubilising structures with lipid uptake receptors [4]. This was confirmed by Yeap et al., who studied the absorption of the poorly water soluble drug cinnarizine solubilised in micelles or vesicles with matched thermodynamic activity (i.e., equal C_free_) in rats [25]. They concluded that ongoing dilution with bile might induce supersaturation and promote absorption. After intake of a meal, complex colloidal structures (i.e., mixed-micelles, (multi)-lamellar vesicles) are formed due to stimulation of bile and pancreatic secretions and the presence of lipid digestion products such as MAGs, FFAs and phospholipids from the meal [18]. At first, mainly lipid droplets and vesicles are present. Upon digestion, the solubilizing capacity of the GI fluids decreases due to dilution with bile and a gradual loss of lipid content within these colloidal structures. The shift from lipid droplets and vesicles to bile salt-phospholipid mixed-micelles promotes transport of solubilised lipophilic compounds across the unstirred water layer to the absorptive surface of the intestine. In addition, the loss of solubilizing capacity might generate periods of supersaturation, enhancing drug absorption [18]. 

When lipolysis is (partially) inhibited by orlistat, the lipid content in the intestinal tract is maintained for a longer time. This sustains solubilisation but hampers the assumed mechanism of bile-induced supersaturation, possibly resulting in a lower absorptive flux. In the present study, however, the increased systemic AUC_0–8h_ in 5 out of 6 volunteers suggests that sustaining fenofibrate solubilisation within the lipid layer is more important than expected. This might indicate that the fast lipolysis of the liquid meal in the condition without orlistat causes not only supersaturation but also precipitation of fenofibrate. Inhibition of lipolysis also influences the intraluminal ultrastructure as less lipid digestion products can be incorporated in mixed-micelles, which shuttle solubilised drug towards the unstirred water layer for absorption. Potentially, the different ultrastructure causes less entrapment of fenofibrate, which would result in an enhanced absorptive flux. Unfortunately, the limited volumes of aspirated jejunal fluids did not allow for determining either intestinal precipitation or supersaturation of fenofibrate or changes in intestinal ultrastructure of the micellar layer. In any case, the observations made in our study warrant more research into the different mechanisms underlying lipophilic drug absorption from fed state media at different levels of digestion.

To exert its action as lipase inhibitor, orlistat needs to be solubilised within the lipid fraction of the meal. It has been shown that the type of meal is important for the extent of duodenal lipolysis inhibition by orlistat [14]. Although HPL was inhibited to the same extent after intake of a solid or liquid meal, the hydrolysis rate of the TAGs from the liquid meal was found to be faster as the TAGs were pre-emulsified in the liquid meal compared to the solid meal. In other words, HPL acted faster on the liquid meal than orlistat on the enzyme. It is plausible that the effect of orlistat on lipolysis would have been more pronounced when a solid meal (i.e., the FDA standard breakfast) was given instead of the liquid meal (Ensure Plus) or when orlistat was pre-dissolved in the liquid meal omitting Xenical dissolution. Whether or not this would still result in an increased fenofibrate absorption remains unclear. 

## 4. Conclusions

Increased solubilisation of lipophilic drugs in fed state GI fluids may or may not result in enhanced drug absorption. The in vivo interplay between drug solubilisation, lipid phase digestion and drug absorption is complex and remains poorly understood. Therefore, this study aimed to investigate the role of fed state GI lipolysis on the absorption of fenofibrate following intake of the LBF Fenogal in healthy volunteers. Inhibition of lipolysis by orlistat resulted in a faster onset of absorption in 4 out of 6 healthy volunteers, reflected by a decrease in systemic T_max_. The increase in intact lipids in the small intestinal tract upon co-administration of the lipase inhibitor orlistat appeared to sustain fenofibrate solubilisation for a longer period and improve absorption, resulting in an increased systemic AUC_0–8h_ in 5 out of 6 volunteers. 

Overall, the obtained in vivo data highlight the importance of including lipids in media used to simulate the impact of fed state conditions on solubilisation and absorption of lipophilic compounds. Ideally, different stages of lipid digestion are considered as this influences the solubilizing capacity of the media and, hence, the solubility-permeability interplay.

## Figures and Tables

**Table 1:** Pharmacokinetic parameters of fenofibric acid in the systemic circulation after intake of Fenogal (200 mg of fenofibrate) in the fed state with and without orlistat.

	T_max_ (min)		C_max_ (µM)		Lag Time to Reach 5% of C_max_ (min)		AUC_0–8h_ (µM.min)	
**Healthy Volunteer**	**− Orlistat**	**+ Orlistat**	**− Orlistat**	**+ Orlistat**	**− Orlistat**	**+ Orlistat**	**− Orlistat**	**+ Orlistat**
HV1	480	340	25.0	31.8	200	60	5306	7809
HV2	420	320	21.4	30.7	140	120	3051	8570
HV3	340	340	36.4	33.7	200	120	7167	8940
HV4	340	320	24.6	44.3	140	200	5798	9882
HV5	360	390	34.3	23.5	220	220	5866	4043
HV6	180	120	25.9	26.2	90	30	6448	8184
Median (range)	350 (180–480)	330 (120–390)	25.5 (21.4–36.4)	31.3 (23.5–44.3)	170 (90–220)	120 (30–220)	5832 (3051–7167)	8377 (4043–9882)

## Data Availability

Data is contained within the article.
